# Membrane-free Electrocatalysis of CO_2_ to C_2_ on CuO/CeO_2_ Nanocomposites

**DOI:** 10.3389/fchem.2022.915759

**Published:** 2022-06-08

**Authors:** Yangming Tian, Xiang Fei, Hui Ning, Wenhang Wang, Xiaojie Tan, Xiaoshan Wang, Zhengguang Ma, Zhihao Guo, Mingbo Wu

**Affiliations:** College of Chemical Engineering, College of New Energy, Institute of New Energy, State Key Laboratory of Heavy Oil Processing, China University of Petroleum (East China), Qingdao, china

**Keywords:** carbon dioxide, membrane free, electrocatalysis, copper, ceria

## Abstract

Carbon dioxide electroreduction (CO_2_RR) with renewable energy is of great significance to realize carbon neutralization. Traditional electrolysis devices usually need an ion exchange membrane to eliminate the interference of oxygen generated on the anode. Herein, the novel CuO/CeO_2_ composite was facilely prepared by anchoring small CuO nanoparticles on the surface of CeO_2_ nanocubes. In addition, CuO(002) crystal planes were induced to grow on CeO_2_(200), which was preferable for CO_2_ adsorption and C-C bond formation. As the catalyst in a membrane-free cell for CO_2_RR, the Cu^+^ was stabilized due to strong interactions between copper and ceria to resist the reduction of negative potentials and the oxidation of oxygen from the counter electrode. As a result, a high Faradaic efficiency of 62.2% toward C_2_ products (ethylene and ethanol) was achieved for the first time in the membrane-free conditions. This work may set off a new upsurge to drive the industrial application of CO_2_RR through membrane-free electrocatalysis.

## Highlight

• Nano CeO2 cube was applied as supports for highly dispersed CuO nanoparticles.
• The CuO(002) crystal faces were preferentially grown on CeO2(200).
• The Faradaic efficiency of CO_2_ to C_2_ exceeds 62% in a membrane-free cell.

## Introduction

Carbon dioxide electroreduction reaction (CO_2_RR) has attracted worldwide attention because of its promising application in suppressing carbon dioxide emission (Gong et al., 2019; Fan et al., 2020; Li H et al., 2021; Sun et al., 2021; Zhao et al., 2021; Li et al., 2022). However, it still needs high performance catalysts to accelerate the conversion of carbon dioxide and control the selectivity of products for the industrial application.

On different catalysts, various products may be obtained, such as carbon monoxide, formic acid, methanol, methane, ethylene, ethanol, oxalic acid, and acetic acid. Among them, the C_2_+ products were desired because of their much higher added value and more important theoretical significance of understanding C-C bond formation (Ma et al., 2020; Li Z et al., 2021; Zhang et al., 2021). Among all the elements, copper is considered as the only candidate as a high selective catalyst for C_2_+ products. In order to achieve better catalytic performance of copper-based catalysts, tremendous works have been carried out from the aspect of crystal faces (Suen et al., 2019), size (Wang et al., 2019), morphology (De Luna et al., 2018), defects (Gu et al., 2021), valence state (Mistry et al., 2016), and surface modification (Li F et al., 2020). Unfortunately, nearly all the pioneer works are executed in a two-chamber cell system, which need a cation or anion exchange membrane to separate the anode and cathode chambers. From a practical and industrial point of view, membranes make the CO_2_RR reactor more complex, increase cost, and limits large-scale application. Therefore, the design of a membrane-free electroreduction process is of great significance. Though with more concise and compact structure, the membrane-free cell still faces several critical challenges, such as the separation of products and the coupling with anodic reaction. For instance, the water oxidation reaction is inevitably on the anode when using aqueous electrolyte. In this condition, the oxygen produced and dissolved in the electrolyte may oxidize the active sites and lead to the decomposition of products in the liquid phase. In order to improve the oxygen tolerance of catalysts for CO_2_RR, several interesting works have been reported (Lu et al., 2019; He et al., 2020). For example, Sun et al. (Li P et al., 2020) developed a CO_2_-selective layer by introducing aniline into the pores of a microporosity polymer to permeate CO_2_ from O_2_ mixture. It was found that the acid–base interaction between CO_2_ and aniline enhances CO_2_ separation from O_2_. Even if the polymer coating is a feasible strategy to protect the active sites from interacting with oxygen, it is not conducive to the effective exposure of active sites. Therefore, new strategies need to be developed to achieve oxygen tolerance and full exposure of active sites simultaneously.

In addition to oxidation, the reduction of active sites in the CO_2_RR process also needs to be considered, whether there is a membrane or not. Especially for the oxide-derived copper (OD-Cu) catalysts, the Cu^+^ has been proposed as the most important active site in the formation of C-C bond, while its stability has always been a great challenge due to its easy reduction under negative applied potentials in the CO_2_RR procedure (Yuan et al., 2018). The strong metal–support interactions have been proved a promising way to improve catalytic activity of metal active sites, which is also applied to upgrade the catalytic performance of copper-based catalysts for the CO_2_RR (Geioushy et al., 2017). For example, Lee et al. (2019) sintered the CuO nanocrystals onto CeO_2_ and found that the ceria is proposed to weaken the hydrogen binding energy of adjacent Cu sites to stabilize the *OCCO intermediate *via* an additional chemical interaction with an oxygen atom in the *OCCO. Zheng et al. (Wang et al., 2018) designed single-atomic Cu–substituted mesoporous CeO_2_ nanorods with multiple oxygen vacancy bond. They found each Cu atom substituted in the CeO_2_(110) surface can be stabilized by coordinating with three oxygen vacancies, yielding a highly effective catalytic center for CO_2_ adsorption and activation to methane. On the contrary, Qiao et al. (Wu et al., 2018) proved the isolated cuprous ions doped in ceria nanorods exhibit strong capability to capture CO_2_ and CO molecules and decrease the energy barrier for producing C_2_ products, leading to a high Faradaic efficiency (FE) of 47.6% toward ethylene. On the other hand, the stabilization effects of ceria substrate toward cuprous ions are responsible for the long-term durability. Sun et al. (Chu et al., 2020a) found that the selectivity and activity of the CO_2_RR on Cu/CeO_2_ composites are depended strongly on the exposed crystal facets of CeO_2_. By tuning the CuO/CeO_2_ interfacial interaction, they further improved the selectivity of ethylene by stabilizing Cu^+^ at the CuO–CeO_2_ interface (Chu et al., 2020b). Han et al. (Yan et al., 2021) modified copper oxide with cerium oxide to enhanced water activation and accelerate the formation rate of *CHO, thus enhance the selectivity and activity of C_2+_ products by promoting the hydrogenation of *CHO. Though the existing works have proved that CeO_2_ is a promising candidate as the supports of copper in the traditional H-cell (with a membrane to separate two champers) for the CO_2_RR process, few works have been reported achieving a high selectivity toward C_2_ products in a membrane-free cell.

Herein, we synthesized a series of novel CuO/CeO_2_ composites by dispersing CuO nanoparticles (NPs) on CeO_2_ nonocubes (NCs), which is then used as catalysts for the CO_2_RR in a single chamber cell without membrane. Surprisingly, the Faradaic efficiency of C_2_ was found exceeding 62% for the first time. This new finding may set off a new research climax in a simple membrane-free cell for industrial perspective of CO_2_ electrocatalysis.

## Results and Discussion

**FIGURE 1:**
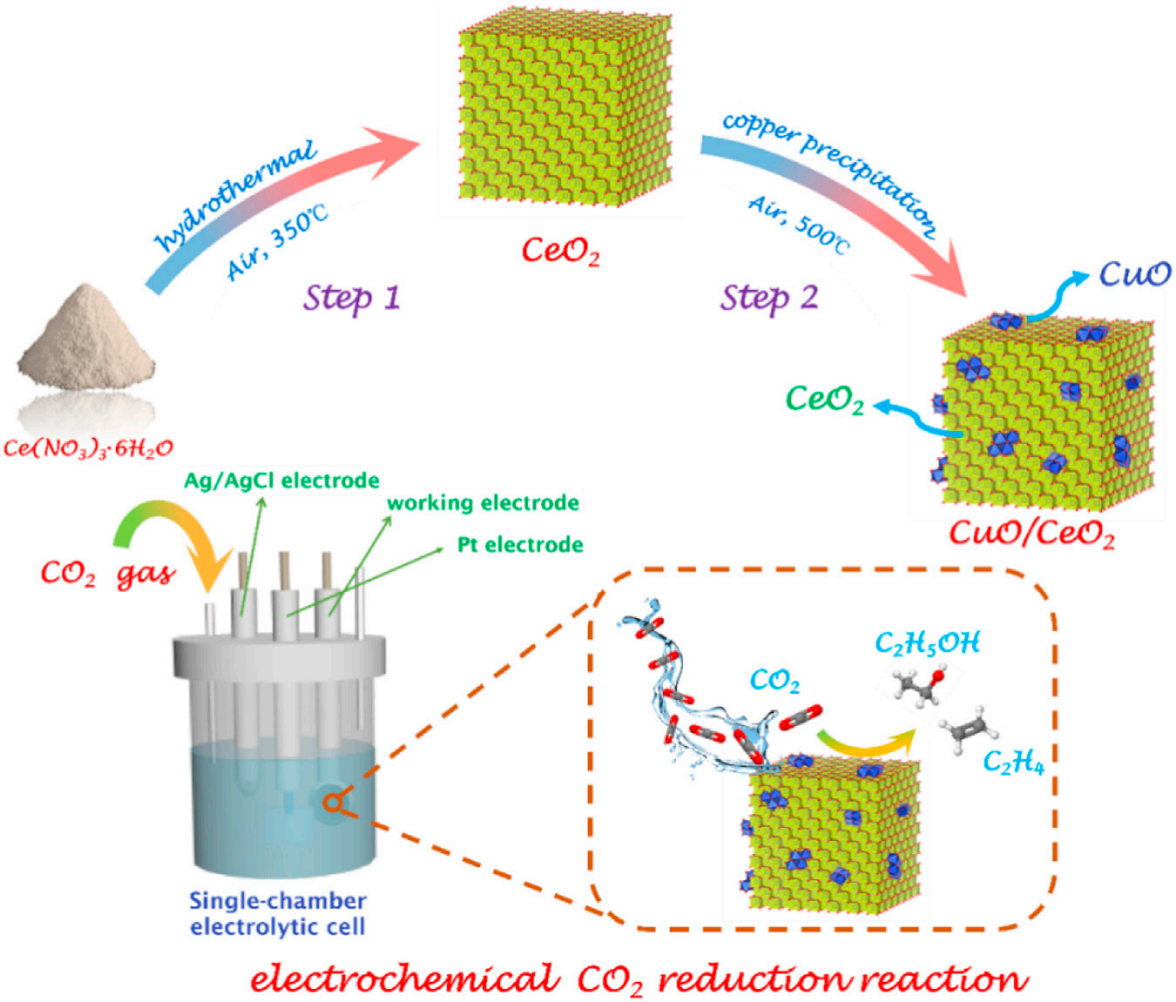
Illustration of the synthesis of CeO_2_ and CuO/CeO_2_.

The fabrication of CuO/CeO_2_ is illustrated in Figure 1. The CeO_2_ NCs were synthesis by a modified hydrothermal method (Zhu et al., 2020). Through an impregnation–precipitation process, copper (II) ions were introduced into ceria lattice. After annealing at 773 K for 3 h, the CuO/CeO_2_ samples were obtained. The detailed composition of CuO/CeO_2_ composites is listed in Supplementary Table S1.

**FIGURE 2:**
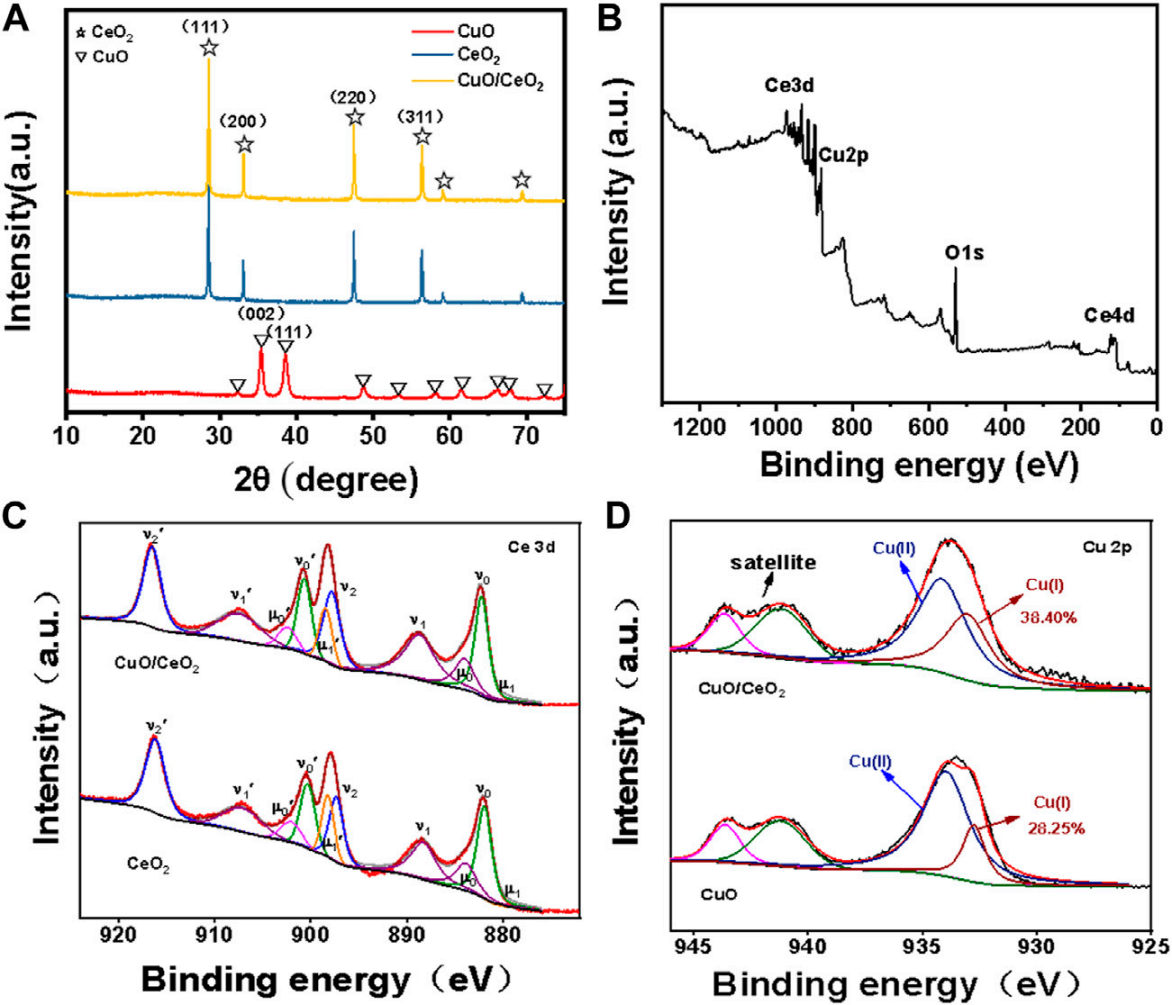
**(A)** XRD spectra of CuO, CuO/CeO_2_ and CeO_2_; **(B)** XPS survey spectra of CuO/CeO_2_; **(C)** XPS of Ce 3d spectra of CuO/CeO_2_ and CeO2; **(D)** XPS of Cu 2p spectra of CuO and CuO/CeO_2_.

The crystal structure of samples as made was firstly investigated by XRD. As depicted in Figure 2A, the characteristic peaks of 28.6°, 33.1°, 47.5°, and 56.4° were ascribed to the (111), (200), (220) and (311) planes of CeO_2_, which is accurately corresponds to the standard spectrum of CeO_2_ (JCPDS No.03-065-0459, space group: Fd-3m Ia3̅, a = 10.774 Å). As a control experiment, we also synthesized CuO following the same procedure but in the absence of CeO_2_. The peaks at 38.8° was corresponded to the (111) planes of CuO (JCPDS No.80-1916). Interestingly, no characteristic peaks of CuO or Cu_2_O were found in the CuO/CeO_2_ composites, which need further characterization.

The detailed element composition of CuO/CeO_2_ was analyzed by XPS spectrum. In Figure 2B, the characteristic peaks of Cu, Ce, and O elements were observed clearly, indicating that the CuO/CeO_2_ composites were successful prepared. The high-resolution XPS spectrum of Ce elopement is shown in Figure 2C. The Ce 3d curve was fitted to 10 peaks corresponding to Ce 3d_5/2_ (μ_1_, 880.5 eV; ν_o_, 882.2 eV; μ_0_, 884.1 eV; ν_1_,888.7 eV; and ν_2_, 897.8 eV) and Ce 3d_3/2_ state (μ_1_′, 898.4 eV; ν_0_′, 900.7 eV; μ_0_′, 902.5 eV; ν_1_′, 907.5 eV; and ν_2_′, 916.6 eV). The peaks of μ_0_, μ_1_, μ_0_′, and μ_1_′ are attributed to Ce^3+^ species, and the remaining peaks are attributed to the Ce^4+^ species (Wang et al., 2018). According to the peak area ratio of the Ce 3d region of CuO/CeO_2_, the relative Ce^3+^ percentage in CeO_2_ is 17.6%, which is much less than that in the pure CeO_2_ (20.8% of Ce^3+^). This phenomenon is derived from the substitution of Ce ions by Cu ions during the synthesis procedure (Wu et al., 2018). The Cu 2p spectra of CuO/CeO_2_ and CuO show distinct copper oxide features in Figure 2D. In addition to the satellite peaks of Cu^2+^, the binding energies (BEs) of 934.2 eV in the Cu 2p_3/2_ region can be attributed to the Cu^2+^ and the peak at 932.7 eV is attributed to the Cu^+^ (Lei et al., 2020) According to the peak area ratio of copper oxidation states in the Cu 2p_3/2_ region, the relative Cu^+^ percentage of CuO dispersed on CeO_2_ is determined to be 38.4%, while the relative Cu^+^ percentage of pure copper oxide is 28.3%. The increased content of Cu^+^ was derived from the transfer of electrons from Ce^3+^ to Cu^2+^ (Chu et al., 2020b).

**FIGURE 3:**
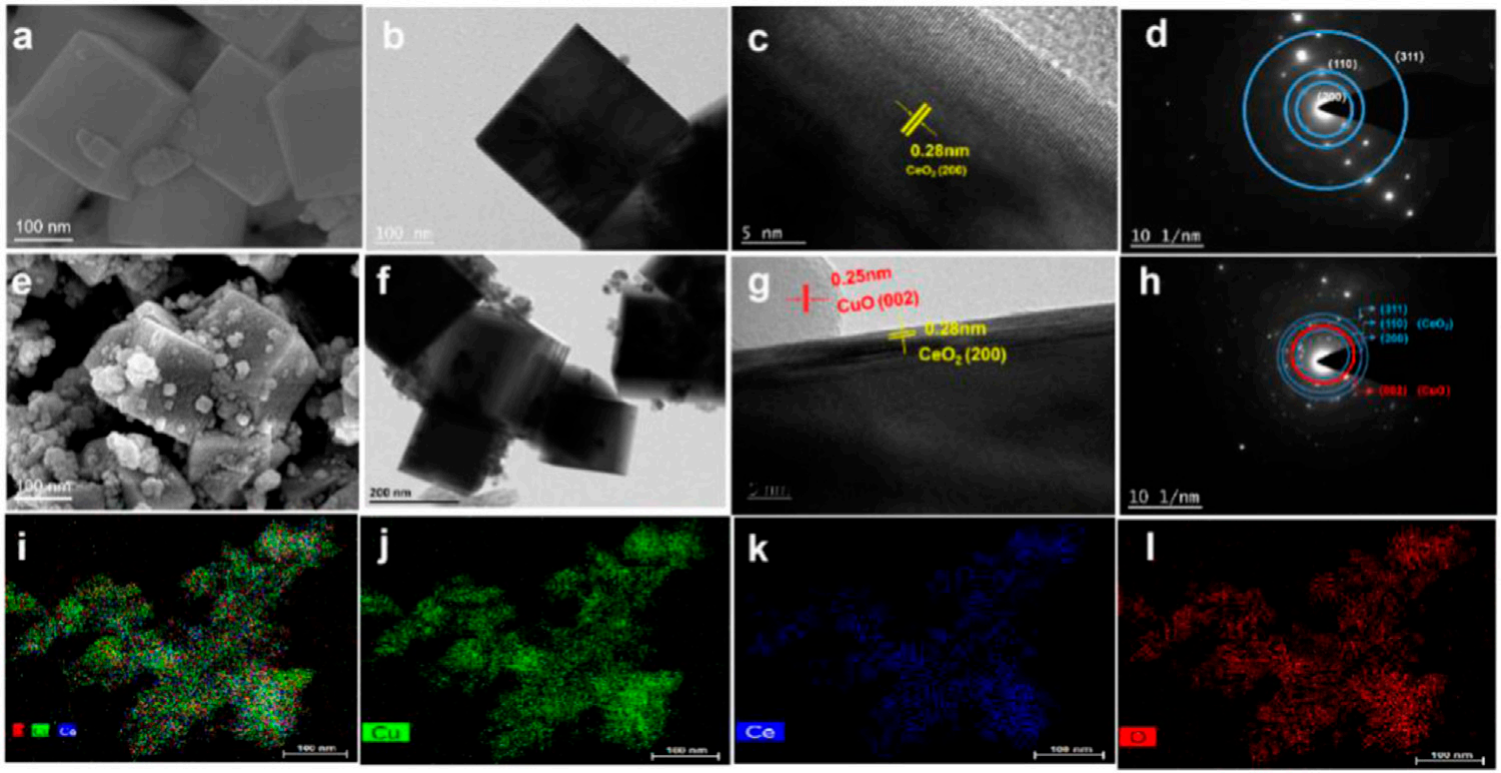
**(A)** SEM images of CeO_2_; **(B)** TEM images of CeO_2_; **(C)** HRTEM images of CeO_2_; **(D)** SAED pattern of CeO_2_; **(E)** SEM images of CuO/CeO_2_; **(F)** TEM images of CuO/CeO_2_; **(G)** HRTEM images of CuO/CeO_2_; **(H)** SAED pattern of CuO/CeO_2_; **(I–L)** EDS mapping of CuO/CeO_2_.

The morphology of CuO/CeO_2_ was verified by the SEM and TEM images. As shown in Figure 3A, the CeO_2_ NPs obtained in this work have an obviously cubic morphology with smooth surface, which size is ca. 23 nm. The morphology of CeO_2_ was confirmed by TEM (Figure 3B) to be classical cubes with clear boundaries. In the HRTEM image, the CeO_2_(200) crystal planes were mainly exposed (Figure 3C), which is confirmed by the SAED pattern (Figure 3D). After compositing with CuO, the cubic morphology and size of CeO_2_ was maintained but the smooth surface was mostly covered by the irregular CuO NPs with the diameter from 20 to 30 nm (Figures 3E,F).

It is worth noting that there is a clear boundary between CuO(002) and CeO_2_(200), as shown in Figure 3G. Furthermore, the SAED pattern of CuO/CeO_2_ (Figure 3H) confirmed that the CeO_2_(200) and CuO(002) planes exist simultaneously and the EDS mapping (Figures 3I–L) indicates the CuO NPs were uniformly dispersed on CeO_2_.

**FIGURE 4:**
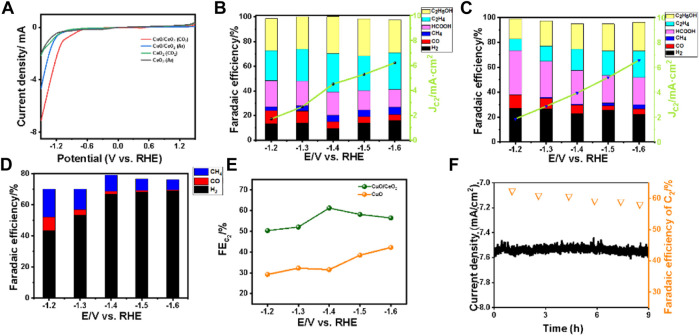
**(A)** LSV curves of CeO_2_ and CuO/CeO_2_ under Ar- and CO_2_-saturated 0.1 M KHCO_3_ in a single cell; **(B)** Faradaic efficiency and C_2_ products current density of CuO/CeO_2_; **(C)** Faradaic efficiency and C_2_ products current density of CuO; **(D)** Faradaic efficiency of CeO_2_; **(E)** Comparison of Faraday efficiency of CuO/CeO_2_ and CuO at different voltages; **(F)** stability curve and Faradaic efficiency of C_2_ during 9 h.

To verify the catalytic performance of the catalysts as made, the LSV curves of all the samples in 0.1 M KHCO_3_ with Ar- and CO_2_-saturated were recorded, as shown in Figure 4A. For the CuO/CeO_2_, larger current density was obtained in CO_2_-saturated 0.1 M KHCO_3_ than in the Ar-saturated conditions, indicating the CuO/CeO_2_ composites have obviously catalytic activity toward CO_2_RR (Chu et al., 2020b). As a control example, the LSV curves of CeO_2_ almost coincide in the Ar- and CO_2_-saturated conditions, indicating the ceria has no activity toward CO_2_RR.

Furthermore, the comprehensive catalytic performance of CO_2_RR on CuO/CeO_2_ under different applied potentials was investigated in a home-made single chamber electrolytic cell without membrane (Supplementary Figure S1). The Faradaic efficiencies of all the products were recorded in Figures 4B–D. Only a small amount of CO and methane were detected in the gas products, which means the CeO_2_ as catalyst has low activity toward CO_2_ electroreduction. While for the CuO/CeO_2_ samples, various products were detected by chromatography, including CO, CH_4_, C_2_H_4_, C_2_H_5_OH, and HCOOH. Take C_2_ (C_2_H_4_ + C_2_H_5_OH) as the target product, as shown in Figures 4C,F, the maximum Faraday efficiency of C_2_ always appears at −1.4 V vs. the reversible hydrogen electrode (RHE) and the FE of C_2_ arrived at the highest value of 62.2%. As control experiments, the maximum FE of C_2_ on pristine CuO under −1.4 V vs. RHE is only 40%, indicating the CeO_2_ as supports can increase the selectivity of CuO toward CO_2_ electroreduction to C_2_. For comparison, the FEs of products on the mixture of CuO and CeO_2_ (with the same ratio of Cu/Ce as CuO/CeO_2_) were also recorded and depicted in Supplementary Figure S3. The FE of C_2_ is 51.1% at −1.4 V vs. RHE, which is lower than that of CuO/CeO_2_ but higher than that of CuO, proving that the presence of CeO_2_ is conducive to the selectivity of C_2_ while the composited state of CuO and CeO_2_ has better performance in producing C_2_ products than the mixed state. Next, the partial current density was applied to evaluate the formation rate of target products. Specially at −1.4 V vs. RHE, the partial current density of C_2_ is 4.5 mA cm^−2^ on CuO/CeO_2_, which is higher than that on CuO (3.9 mA cm^−2^). While in the H-type cell, with the optimum CuO/CeO_2_ catalyst, the FE of C_2_ is 58.4% with a partial current density of 4.3 mA cm^−2^, as shown in Supplementary Figure S4. The higher selectivity and activity further prove the superiority of our catalyst in a single cell than the H-type cell.

We also tested the long-time durability of different catalysts in the membrane-free condition (Figure 4F and Supplementary Figure S5). After 9 h’ electrocatalysis at −1.4 V vs. RHE, the catalytic performance of CuO/CeO_2_ is stable with no significant change and the Faradaic efficiency for C_2_ remains around 60%. On the contrary, when the pristine CuO was used as catalyst, the current density had a noticeable increasing over time and the FE of C_2_ decreased from 40 to 21%, which mean the Cu^+^ in CuO cannot be stabilized and the selectivity of C_2_ was synchronously reduced.

**FIGURE 5:**
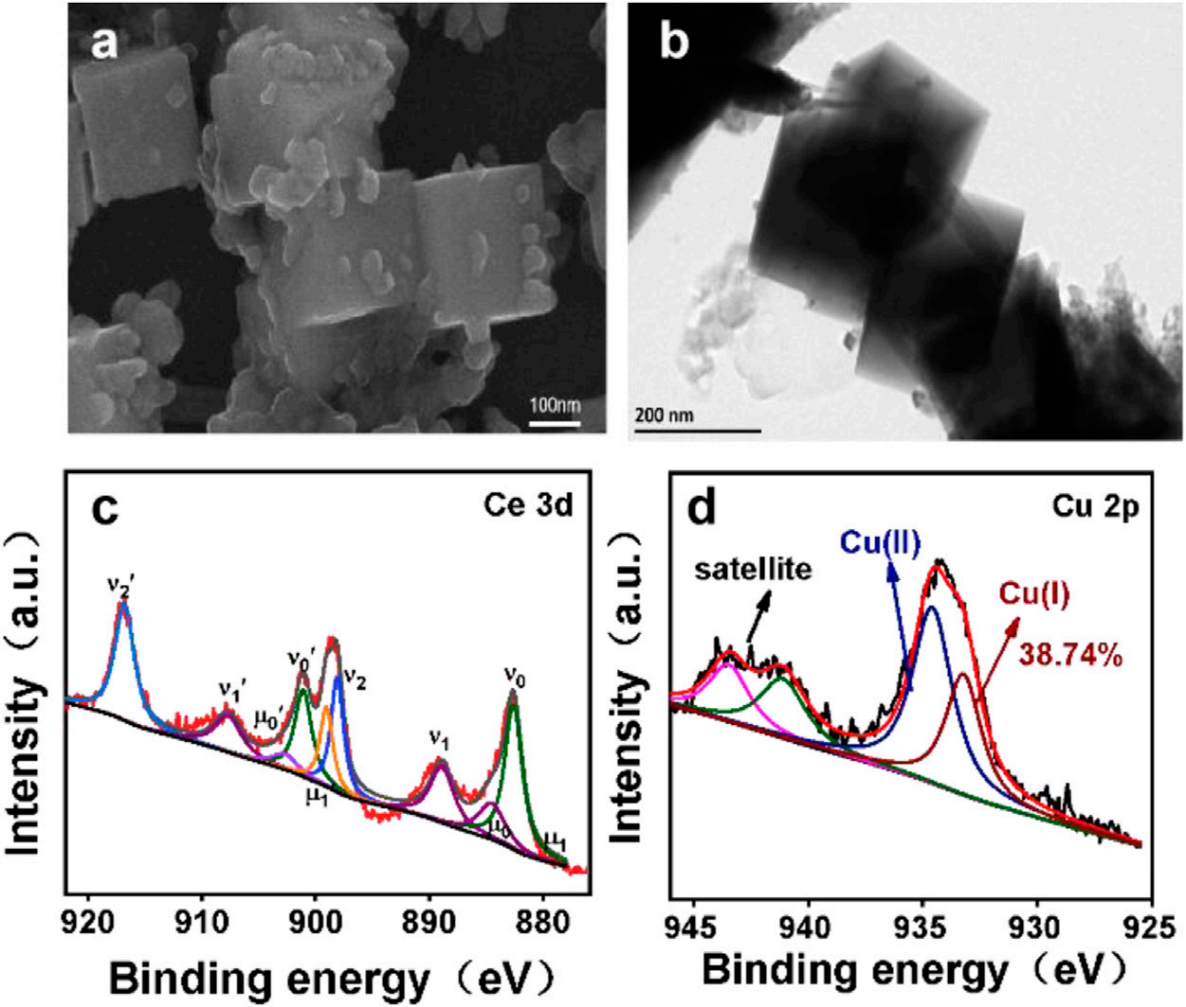
**(A)** SEM images of CuO/CeO_2_; **(B)** TEM images of CuO/CeO_2_; **(C)** XPS of Ce 3d spectra of CuO/CeO_2_; **(D)** Cu 2p spectra of CuO/CeO_2_ after CO_2_RR.

In order to unveil the reason of different performance between CuO/CeO_2_ and pristine CuO, the composition and morphology of catalyst after electrocatalysis were characterized. As proved by SEM and TEM image in Figures 5A,B, the morphology of CuO/CeO_2_ was well maintained after reaction. In addition, the XPS results further revealed that the ratio of Cu^+^/(Cu^+^ + Cu^2+^) has no significant change compared to the fresh catalyst, indicates Cu^+^ can be well stabilized by CeO_2_ during the CO_2_RR process. The stability of the morphology and valence state of the CuO/CeO_2_ composite further proves that the stabilization effects of ceria toward Cu^+^, which provides favorable catalysts for the high-efficiency electroreduction of carbon dioxide in a membrane-free electrolyzer.

## Conclusion

In this work, the CeO_2_ NCs with average size of 230 nm were facilely synthesized by a hydrothermal method. Further through an impregnation–calcination process, the CuO NPs with diameters from 20 to 30 nm was highly dispersed on the surface of CeO_2_ to fabricate novel composites of CuO/CeO_2_, in which the mainly exposed crystal faces was CuO(002) and CeO_2_(200). Due to the strong metal–metal oxide interaction between copper and ceria, the content of Cu^+^ was increased in the CuO/CeO_2_ composite and stabilized in the CO_2_RR process, which makes the CuO/CeO_2_ an excellent catalyst for CO_2_RR toward C_2_ products with high selectivity and stability in a single cell without membrane. This study opens up a new way to realize the high-efficiency electroreduction of CO_2_ by using a simple membrane-free system.

## Data Availability

The original contributions presented in the study are included in the article/Supplementary Material; further inquiries can be directed to the corresponding authors.
