# Supplementary material for: Membrane-free Electrocatalysis of CO2 to C2 on CuO/CeO2 Nanocomposites
Source: Front Chem. 2022 Jun 8;10:915759. doi: 10.3389/fchem.2022.915759 (PMC9215358; doi:10.3389/fchem.2022.915759)
Supplement: Supplementary file 1 [file DataSheet1.docx]

**Supporting Information**

**Membrane-free electrocatalysis of CO_2_ to C_2_ on CuO/CeO_2_ nanocomposites**

Yangming Tian ^a^, Xiang Fei ^a^，Hui Ning ^a,*^, Wenhang Wang ^a^, Xiaojie Tan ^a^ , Xiaoshan Wang ^a^，Zhengguang Ma ^a^，Zhihao Guo ^a^, Mingbo Wu ^a,*^

^a^ College of Chemical Engineering, College of New Energy, Institute of New Energy, State Key Laboratory of Heavy Oil Processing, China University of Petroleum (East China), Qingdao 266580.

*Corresponding authors：

Prof. Hui Ning, E-mail: ninghui@upc.edu.cn

**Experimental section**

**Materials**

All chemicals and reagents were purchased from Sinopharm Chemical Reagent Co., Ltd. Unless otherwise specified. The water was purified by a Millipore system in all experiments. High purity Ar (99.999%), N_2_ (99.999%) and CO_2_ (99.999%) were obtained by Qingdao Xinkeyuan technology Co., Ltd.

**Synthesis of CuO/CeO_2_**

The fabrication of CuO/CeO_2_ were illustrated in Fig. 1. The CeO_2_ nanocubes were synthesis by a hydrothermal method ^1^. Ce(NO_3_)_2_·6H_2_O was dissolved in the prepared concentrated NaOH solution, and the hydrothermal reaction was carried out at 350 K for 24 h. After centrifugation and washing, the cubic cerium oxide was obtained by high temperature calcination at 623 K. Then, 0.25 g of cubic CeO_2_ was dispersed in 20 mL of deionized water by ultrasonic for 0.5 h. After that, 10 mL of 0.065 mol L^-1^ of Cu(NO_3_)_2_ solution was added into the above suspension and the pH was adjusted to 9-10 with 0.5 mol L^-1^ Na_2_CO_3_ solution. In the end, the precipitate was filtered and calcinated at 773 K for 3 hours to obtain the CuO/CeO_2_ samples.

**Preparation of the mixture of CuO and CeO_2_**

10 mL of 0.065 mol L^-1^ of Cu(NO_3_)_2_ solution was added into 20 mL of deionized water and the pH was adjusted to 9-10 with 0.5 mol L^-1^ Na_2_CO_3_ solution. Then, the precipitate was filtered and calcinated at 773 K for 3 hours to obtain the CuO. After that, the obtained CuO was ground with 0.25g CeO_2_ to obtain the mixture of CuO and CeO_2_.

**Characterizations**

The crystal structure was investigated by X-ray diffraction (XRD, X’Pert PRO MPD, Holland) with Cu Kα (40 kV, 40 mA, λ=1.5406 Å). Field emission scanning electron microscopy (FESEM, Hitachi S-4800, Japan) images and transmission electron microscopy (TEM, JEM-2010, 220 kV, Japan) images were applied to observe the morphology of the materials. The elemental compositions and chemical bonding were analyzed by X-ray photoelectron spectroscopy (XPS, Thermo Scientific Escalab 250XI, America) with Al Kα radiation.

**CO_2_ Electrochemical Measurements**

All of electrochemical tests were tested by diaphragmless-type electrolytic cell (single cell) at room temperature on the electrochemical workstation (CHI 660E, Shanghai CH Instruments Co., China).

In single cell, both anode and cathode are placed in the same cell which were filled with 40 mL of 0.1 M KHCO_3_ aqueous solution as electrolyte. A L-type glassy-carbon (GC, ø = 12 mm) coated with material, Ag/AgCl electrode (saturated KCl solution) and a platinum gauze (1×1 cm^2^) were used as working, reference and counter electrode, respectively. The pH value of the CO_2_ saturated electrolyte is 6.8. All potentials cited were referenced to the reversible hydrogen electrode (RHE) in this work. Before electrochemical testing, the electrolyte was bubbled with N_2_ for 30 min to exclude air in the solution. Then CO_2_ was bubbled into the electrolyte in the cathode side for more than 30 min under stirring until saturated and the CO_2_ gas flow was controlled at a steady stream (20 sccm) during the electrochemical measurements.

Preparation of working electrode: The GC electrode was polished and rinsed thoroughly in water and ethanol, then dried with wiper for lens. 100 µL of catalyst ink (2.0 mg materials in 10 µL of 5% Nafion solution and 200 µL of isopropanol) dripped on the GC electrode and dried with N_2_.

**Product analysis**

The resulting gas phase products in the cathodic side during CO_2_ electrolysis was online tested across gas chromatograph (BFRL-3420A, China) using a ten-port valve system with Ar as carrier gas. The gas chromatograph equipped thermal conductivity detector (TCD) detected hydrogen and flame ionization detector (FID) detected hydrocarbons and carbon monoxide with a methanator. The liquid products were quantified by a high-performance liquid chromatograph (HPLC, LC-2030 Plus, Shimadzu, Japan) with differential refraction detector (RID-20A). All products were quantitatively analyzed by external standard curve method.

**The** **calculation of faradic efficiency**

The catalysts were subjected to i-t tests at different voltages, and the gas-phase products were qualitatively and quantitatively analyzed by chromatography, and then the Faradaic efficiencies of each gas-phase product were calculated separately. The formula is:

FE%=$\frac{p\times{10}^{-6}\times v\times{10}^{-6}\times96485.3\times101300\times\alpha}{8.314\times298.15\times i\times60}$×100%

(**p** is the concentration of the product, **i** is the current, **α** is the number of electrons transferred to produce the product, and **v** is the flow rate of CO_2_.)

The Faradaic efficiency calculation of the liquid phase product is published as:$FE=\frac{e*n*96500}{Q}$

(**e** is the electron transfer number, **n** is the amount of substance in the liquid phase product, and **Q** is the total amount of electricity generated during electrolysis.)

**Figures and Tables**


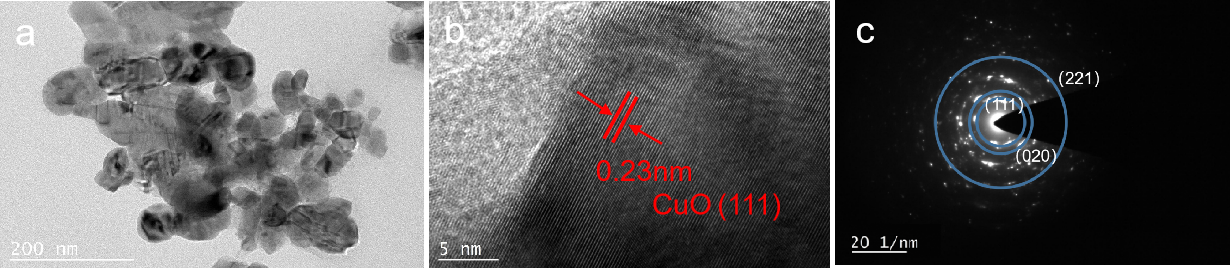


**Figure S1.** TEM and HRTEM images of (a, b) CuO, (c) SAED pattern of the CuO


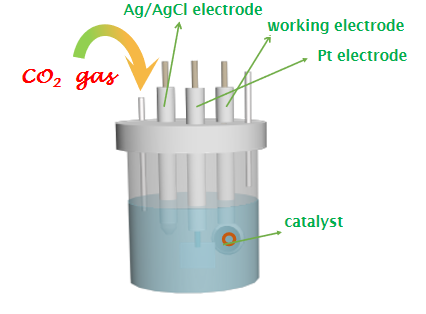


**Figure S2.** Schematic diagram of a single-cell without membrane.

**Figure S3.** The Faradaic efficiencies of products on the mixture of CuO and CeO_2_ in the single cell.

**Figure S4.** The Faradaic efficiencies of products on CuO/CeO_2_ in the H-type cell.


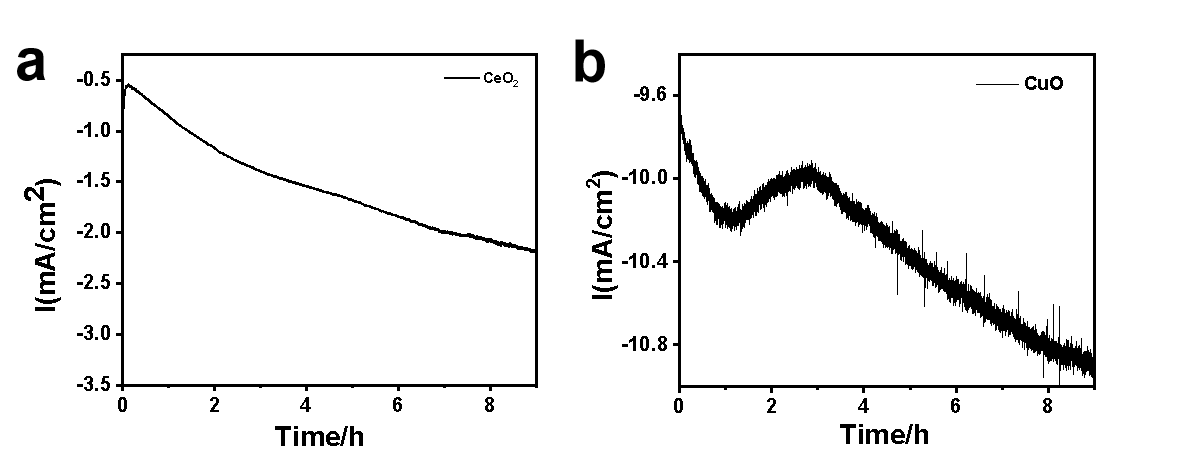


**Figure S5.** The stability tests of (a) CeO_2_ and (b) CuO at −1.4 V (vs. RHE)

**Table S1.** Comparison of the electrocatalytic performance with other copper/ceria materials

| **Catalyst** | **Production** | **FE** | **Potential** | **electrolyte** |
| --- | --- | --- | --- | --- |
| **CuO/CeO_2_** | C_2_ | 61.23% | -1.4 V vs. RHE | 0.1M KHCO_3_ |
| **CeO_2_/CuO^3^** | C_2_ | 75.2% | -1.12 V vs. RHE | 1.0M KOH |
| **Cu-CeO_2_-4%^4^** | CH_4_ | 58% | −1.8 V vs. RHE | 0.1M KHCO_3_ |
| **Cu/CeO_2_(110)^5^** | C_2_H_4_ | 39.1% | -1.13 V vs. RHE | 0.1M KHCO_3_ |
| **CuO-CeO_2_/CB^6^** | C_2_H_4_ | 50% | -1.1 V vs. RHE | 0.1M KHCO_3_ |
| **Cu/ceria NPs (Cu/Ce = 0.95)^7^** | C_2_H_4_ | 49.2% | −1.0 V vs. RHE | 0.1M CsHCO_3_ |

**References**

1. C. Zhu, X. Wei, W. Li, Y. Pu, J. Sun, K. Tang, H. Wan, C. Ge, W. Zou and L. Dong, *ACS Sustainable Chemistry & Engineering*, 2020, **8**, 14397-14406.

2. W. Ma, S. Xie, T. Liu, Q. Fan, J. Ye, F. Sun, Z. Jiang, Q. Zhang, J. Cheng and Y. Wang, *Nature Catalysis*, 2020, **3**, 478-487.

3. X. Yan, C. Chen, Y. Wu, S. Liu, Y. Chen, R. Feng, J. Zhang and B. Han, *Chem Sci*, 2021, **12**, 6638-6645.

4. Y. Wang, Z. Chen, P. Han, Y. Du, Z. Gu, X. Xu and G. Zheng, *ACS Catalysis*, 2018, **8**, 7113-7119.

5. S. Chu, X. Li, A. W. Robertson and Z. Sun, *Acta Physico Chimica Sinica*, 2020, **0**, 2009023-2009020.

6. S. Chu, X. Yan, C. Choi, S. Hong, A. W. Robertson, J. Masa, B. Han, Y. Jung and Z. Sun, *Green Chemistry*, 2020, **22**, 6540-6546.

7. C. W. Lee, S.-J. Shin, H. Jung, D. L. T. Nguyen, S. Y. Lee, W. H. Lee, D. H. Won, M. G. Kim, H.-S. Oh, T. Jang, H. Kim, B. K. Min and Y. J. Hwang, *ACS Energy Letters*, 2019, **4**, 2241-2248.
